# Quantitative calibration of Tb-161 SPECT/CT in view of personalised dosimetry assessment studies

**DOI:** 10.1186/s40658-024-00611-9

**Published:** 2024-02-19

**Authors:** Lachlan McIntosh, Price Jackson, Brittany Emmerson, James P. Buteau, Ramin Alipour, Grace Kong, Michael S. Hofman

**Affiliations:** 1 Prostate Cancer Theranostics and Imaging Centre of Excellence (ProsTIC), Molecular Imaging and Therapeutic Nuclear Medicine, Cancer Imaging, Peter MacCallum Centre, Melbourne, Australia; 2 School of Science, RMIT University, Melbourne, Australia; 3 Sir Peter MacCallum Department of Oncology, University of Melbourne, Melbourne, Australia

**Keywords:** Tb-161, SPECT, QSPECT, Dosimetry

## Abstract

**Background:**

Terbium-161 (^161^Tb)-based radionuclide therapy poses an alternative to current Lutetium-177 (^177^Lu) approaches with the additional benefit of secondary Auger and conversion electron emissions capable of delivering high doses of localised damage to micro-metastases including single cells. Quantitative single-photon emission computed tomography, paired with computed tomography (SPECT/CT), enables quantitative measurement from post-therapy imaging. In view of dosimetry extrapolations, a Tb-161 sensitivity SPECT/CT camera calibration was performed using a method previously validated for ^177^Lu.

**Methods:**

Serial imaging of a NEMA/IEC body phantom with Tb-161 was performed on SPECT/CT with low-energy high-resolution collimators employing a photopeak of 75 keV with a 20% width. Quantitative stability and recovery coefficients were investigated over a sequence of 19 scans with buffered ^161^Tb solution at total phantom activity ranging from 70 to 4990 MBq.

**Results:**

Sphere recovery coefficients were 0.60 ± 0.05, 0.52 ± 0.07, 0.45 ± 0.07, 0.39 ± 0.07, 0.28 ± 0.08, and 0.20 ± 0.08 for spheres 37, 28, 22, 17, 13, and 10mm, respectively, when considered across all activity and scan durations with dual-energy window scatter correction. Whole-field reconstructed sensitivity was calculated as 1.42E−5 counts per decay. Qualitatively, images exhibited no visual artefacts and were comparable to ^177^Lu SPECT/CT.

**Conclusions:**

Quantitative SPECT/CT of ^161^Tb is feasible over a range of activities enabling dosimetry analogous to ^177^Lu whilst also producing suitable imaging for clinical review. This has been incorporated into a prospective trial of ^161^Tb-PSMA for men with metastatic prostate cancer.

**Supplementary Information:**

The online version contains supplementary material available at 10.1186/s40658-024-00611-9.

## Background

Quantitative SPECT/CT imaging provides a method of calculating post-therapy dosimetry from targeted radionuclide therapy which may provide pertinent information in terms of dose–response [1–6]. Terbium-161 (^161^Tb)-labelled targeted radionuclide therapy offers a potential alternative to Lutetium-177 (^177^Lu)-labelled therapeutic agents given its similar decay characteristics in half-life (6.95 vs 6.7 days), beta energy (154 vs 134 keV), and gamma emission (74.6 keV [10.2%] vs 208 keV [10.4%]) suitable for SPECT-CT imaging [7–10]. Most notably, however, ^161^Tb emits additional conversion and Auger electrons capable of providing a significantly higher energy deposition per decay in micro-metastases and single cells, with consequent potential for improved therapeutic efficacy as indicated by Monte-Carlo studies [11–14], and, in pre-clinical studies, with tumour-targeting folate conjugate [15] and prostate-specific membrane antigen (PSMA) [16].

Challenges in production have limited research with ^161^Tb until recent advancements which provided enough activity for clinical applications [17, 18]. These advancements have enabled the initiation of several ^161^Tb-labelled targeted radionuclide therapy clinical trials, including the recent first in-human study with ^161^Tb-DOTATOC that demonstrated the ability to visualise small metastases from SPECT/CT imaging with relatively low administered activity (< 1–5 GBq) [19].

In preparation for the VIOLET trial, a phase I/II clinical trial designed to evaluate the safety and efficacy of ^161^Tb-PSMA-I&T (NCT05521412) in men with metastatic castration-resistant prostate cancer [20], we aimed to assess the image quality and quantitative capabilities of post-therapy SPECT/CT acquisitions and reconstructions over a clinical activity range.

## Methods

Calibrated no-carrier-added ^161^Tb radionuclide sample was obtained from supplier (Isotopia Molecular Imaging, Petah Tikva, Israel). A NEMA IEC body phantom was filled at 8:1 ratio between spheres and background with diethylenetriaminepentaacetic acid used as buffer to prevent potential adsorption to phantom linings. The phantom was imaged over 19 delayed timepoints to determine quantitative stability of recovery coefficients relative to total in-field activity level (70–4990 MBq). All images were acquired on an integrated Siemens Symbia Intevo Bold SPECT/CT scanner (Siemen’s Healthineers, Erlangen, Germany) for 120 frames per rotation and OSEM reconstruction (Flash-3D, 6 iterations, 10 subsets, 6.0mm Gaussian smoothing, CT AC, 4.8 mm^3^ voxel size) using low-energy high-resolution collimators focused on a photopeak of 74.6 keV ± 10% with ± 6% upper and lower scatter window widths (TEW) and lower scatter only (DEW). Reconstruction parameters were chosen based on the work by Marin et al. [10] that found convergence for all NEMA spheres using the same energy window and image updates (subset/iteration configuration). For each study, separate acquisitions with frame durations of 2, 4, 8, and 16 s were performed to evaluate the effects of count rate and reconstruction count density with respect to apparent camera sensitivity. Additionally, the choice of dual-energy window (DEW) or triple-energy window (TEW) scatter correction during reconstruction was evaluated using paired samples t test.

Phantom images were analysed for NEMA sphere contrast-to-noise ratio (CNR), mean volumetric recovery coefficients (RC), and background count sensitivity. NEMA sphere volume-of-interest (VOI) was defined by the known diameter of the spherical inserts of the phantom on an initial CT, drawn using MIM (MIM Software Inc., USA), and propagated across all reconstructed series by automated rigid image registration [21]. Background VOI was defined by two large cylinders (each with 5 cm diameter, 15 cm length, 300 mL volume); one drawn at 2.5 cm axial offset from the 22 mm and 28 mm sphere VOI, and the other drawn at 2.5 cm axial offset from the 10 mm and 37 mm sphere (see Additional file 1: Fig. S1). Whole-field VOI was defined by all voxels in the reconstructed image. Sphere CNR was calculated by subtracting the average counts in the background VOI from the measured mean sphere VOI divided by the background noise (standard deviation). RC was defined by dividing the measured mean activity concentration in the specific sphere VOI by the activity concentration known from the phantom experiment preparation.

Scanner sensitivity was scored as reconstructed counts per Becquerel and second of acquisition (counts/Bq*s). In-field sensitivity was assessed by three methods: total counts in the reconstructed image relative to total activity, background VOI count concentration with respect to activity concentration, and IEC sphere count concentration with relative to 8:1 activity concentration as adjusted by measured RC. Activity values were measured in syringe using a Capintec-CRC 15R dose calibrator with calibration code number #197 based on cross-calibration of supplier quoted activity. Determination of final sensitivity values was obtained by calculation of the mean of observed sensitivity values over a range of clinically expected in-field count densities, ranging from 250 to 2000 MBq. Additionally, count rate effects were evaluated from raw tomographic images in terms of count fractions of upper and lower scatter relative to photopeak window.

Voxel dose kernel for ^161^Tb was calculated using the Geant4 Application for Tomographic Emission (GATE) software [22] and applied to the cumulated activity map derived from serial quantitative ^161^Tb SPECT/CT images acquired at 4, 24, and 120 h post 5.4 GBq administration for a single patient using existing voxel registration and kinetics (VRAK) software [1]. SPECT/CT images were acquired using 10 s frame duration, CT attenuation correction, Flash-3D reconstruction algorithm with 4 iterations, 8 subsets, and 8.40 mm Gaussian post-processing smoothing filter.

## Results

**Fig. 1:**
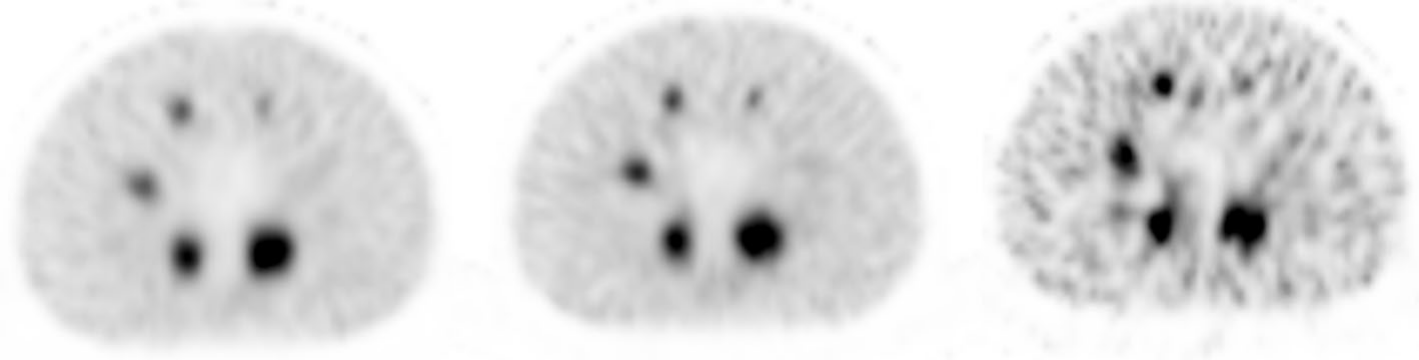
Example axial slices acquired using 16 s frame duration, 60 frames of rotation, and dual-energy window at activity levels 5300 MBq (left), 2200 MBq (middle), 300 MBq (right). Total photopeak counts 38.5E6, 18.9E6, and 2.96E6, respectively

**Fig. 2:**
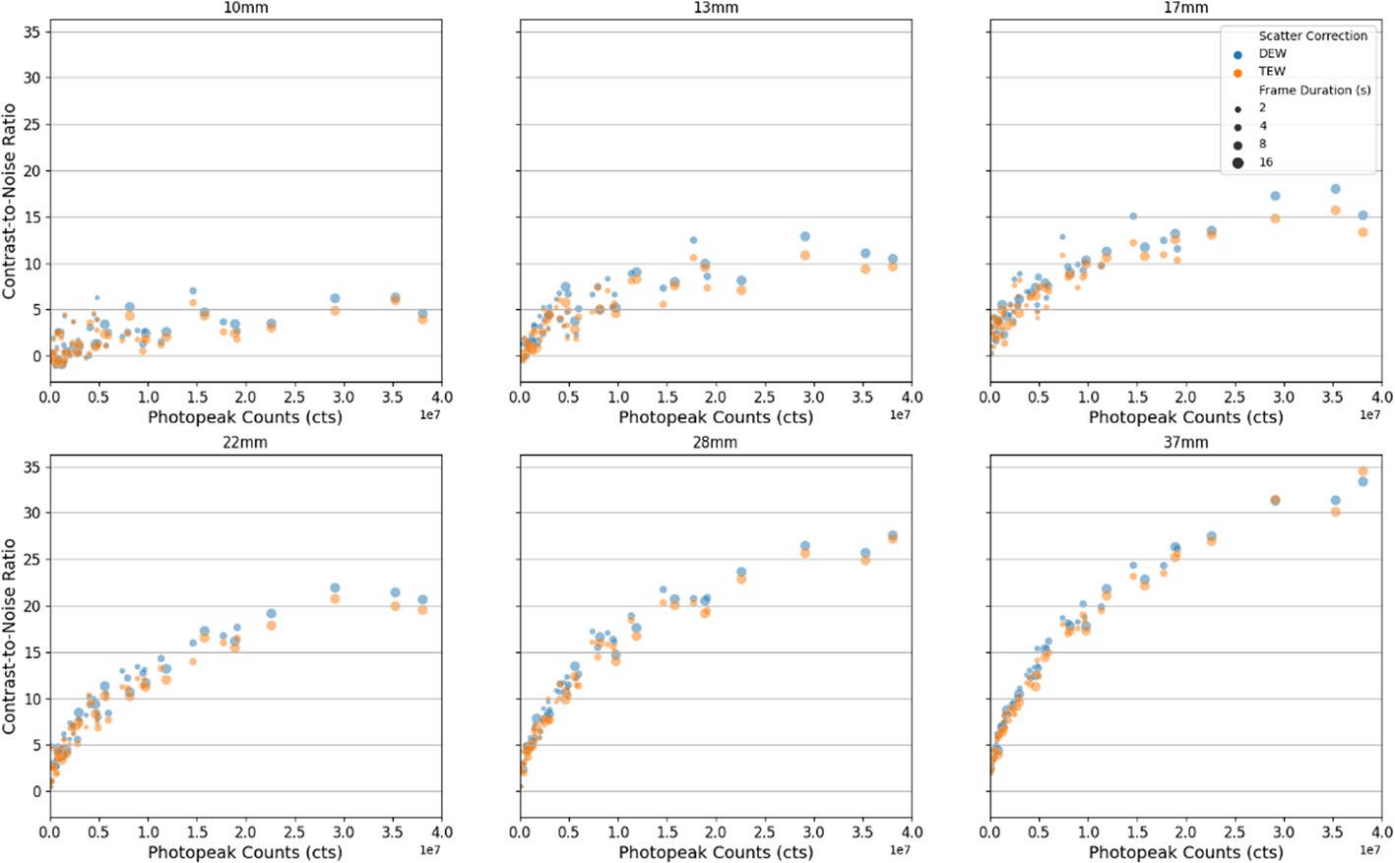
Contrast-to-noise ratio for each NEMA IEC sphere as a function of the acquired statistics (photopeak counts) with dual- and triple-energy window scatter corrections

**Fig. 3:**
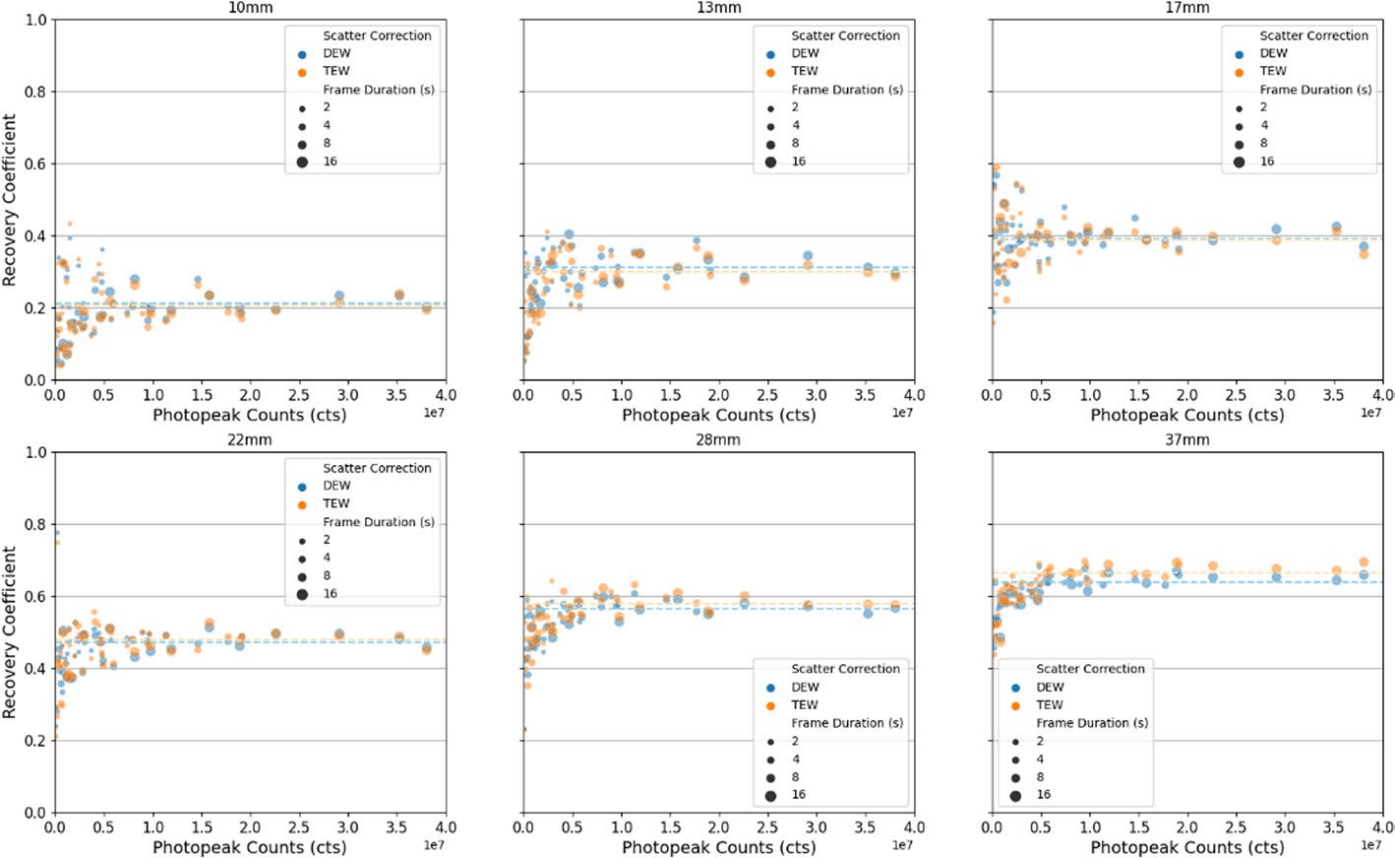
Measured recovery coefficients for NEMA IEC sphere phantom as a function of the acquired statistics (photopeak counts) with dual- and triple-energy window scatter corrections. Each point represents the recovery for the given sphere size at each imaging session over the course of the decayed observations

**Fig. 4:**
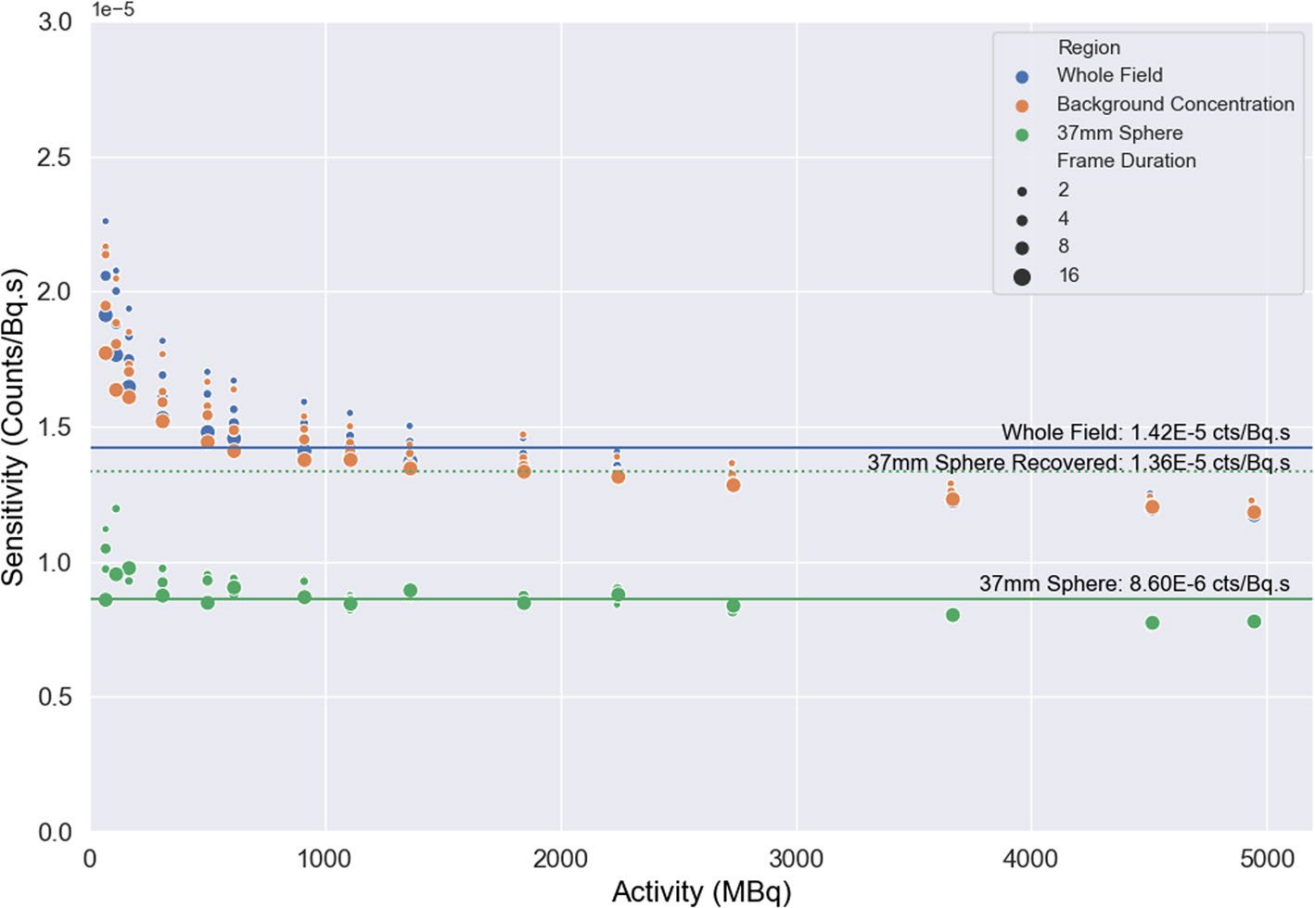
Sensitivity (*y*-axis) as measured for different total ^161^Tb activity quantities (*x*-axis) based on whole-field counts, background, and 37mm sphere concentrations. The effect of frame duration (2–16 s) is illustrated by marker size

Visually acceptable images with clear delineation of the four largest NEMA IEC spheres (22 + mm) were obtainable with total activities as low as 300 MBq (Fig. 1). When > 1e7 photopeak counts were acquired, Rose criterion (CNR > 5) was met for all spheres, excluding 10 mm radius (Fig. 2). Sphere RC were 0.60 ± 0.05, 0.52 ± 0.07, 0.45 ± 0.07, 0.39 ± 0.07, 0.28 ± 0.08, and 0.20 ± 0.08 for spheres 37, 28, 22, 17, 13, and 10mm, respectively, when considered across all activity and scan durations with DEW scatter correction (Fig. 3). Sphere RC were comparable between both DEW and TEW scatter correction methods and neither technique could be characterised with a clear improvement in count linearity with respect to activity (Fig. 3). Uncorrected dead-time effects were observed at very high in-field activities above 2 GBq in single FOV which reduced the apparent sphere sensitivity by up to 20% at 5 GBq; an in-field activity likely to exceed most clinical situations (Fig. 4). Evaluating whole-field sensitivity across a viable range of clinical count densities, from 250 to 2000 MBq with 8 and 16 s frame durations, a sensitivity value of 1.42E−5 cts/Bq*s was determined.

**Fig. 5:**
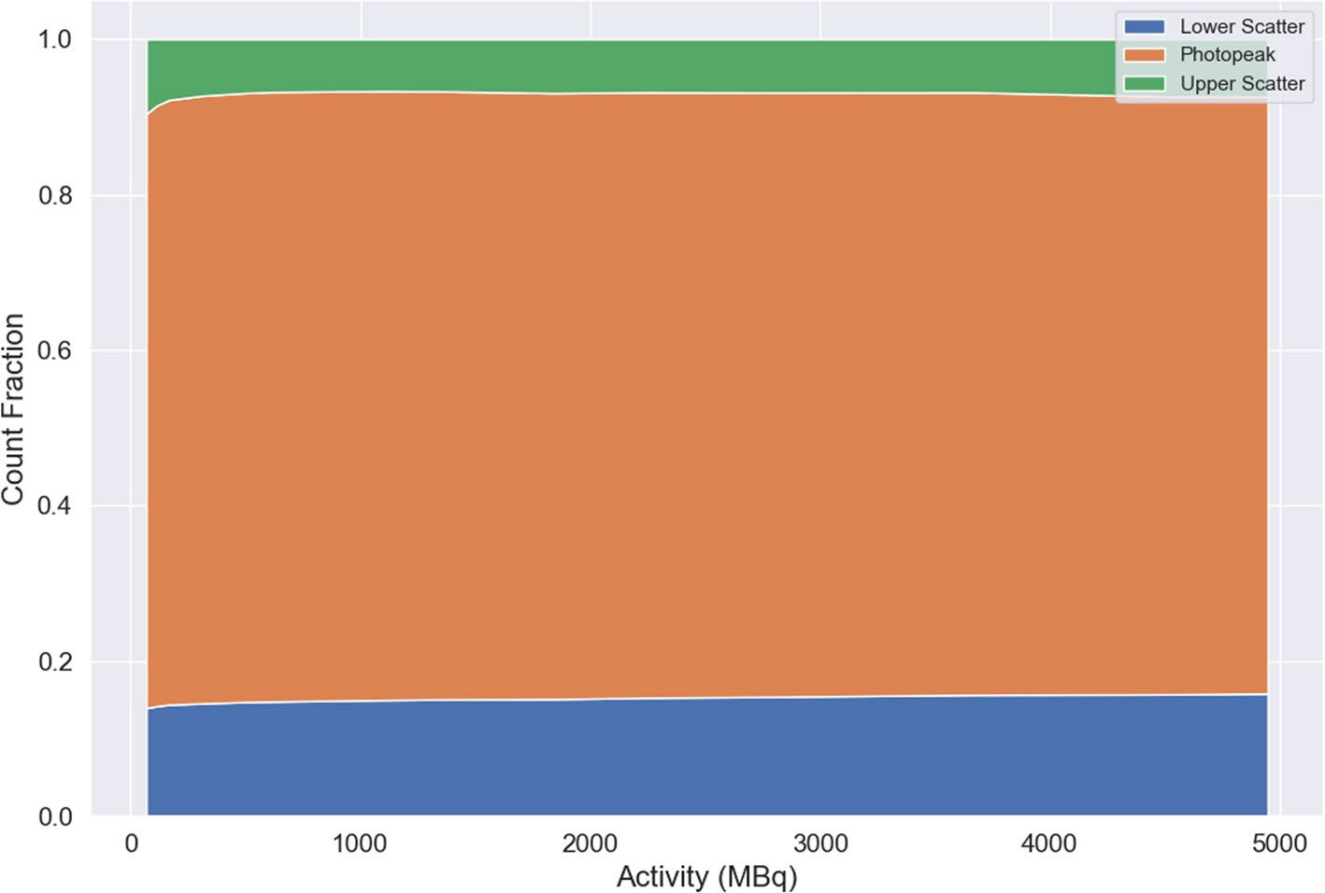
Count fraction in lower scatter (blue), photopeak (orange), and upper scatter (green) windows in raw tomographic dataset with respect to imaged activity

Count rate effects were evaluated in terms of relative counts in photopeak compared to scatter windows and showed no evidence of count pile-up (Fig. 5). Count fractions were consistent across all acquisitions except for very low activity levels. Additional analysis of count rate in projection views was approximately linear with in-field activity across all windows apart from a small dark-field background count rate; in the order of 100 counts per second in the photopeak energy window.

An appreciable increase in background region of interest (ROI) sensitivity was observed in conjunction with reduced local count density on the reconstructed image. The effect was linked to the total acquired statistics, proportional to the total activity present in the FOV and the length of the acquisition (i.e. the length of the frame duration) with higher apparent sensitivity observed at lower activity concentration and acquisition times. Because projection view count rates were approximately linear, this suggests that the variable sensitivity is reconstruction-related; likely owning to high standard deviation of counts relative to the mean of the region. Examining the histogram distribution of background counts across different activity levels and scan durations (Additional file 1: Fig. S2) shows a clear deviation from normal distribution at low count density and may relate to systems avoidance of negative values in the reconstruction process. Additionally, the overall stability of sensitivity values to high-uptake sphere regions suggests that the effect is related to count density and applying the average 37mm sphere RC (0.60) to the observed local sensitivity yielded a comparable value of 1.36E−5 counts/Bq*s.

No visual or statistical (students *t* test) improvement in sphere RC was observed with TEW scatter correction technique (*p* > 0.05), though a lower whole-field sensitivity was observed (1.25E−5 counts/Bq*s).

**Fig. 6:**
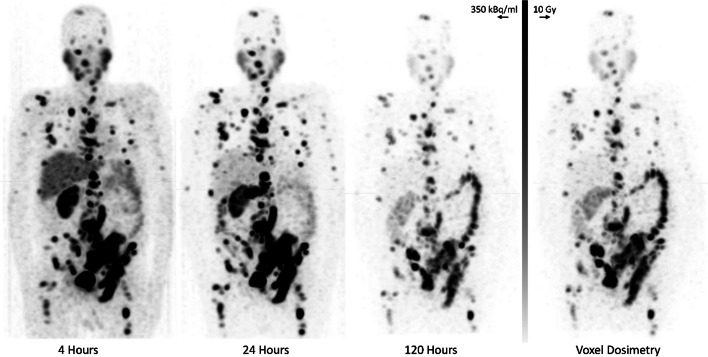
Sequential QSPECT patient imaging following 161Tb-PSMA administration (4, 24, 120 h) and derived dosimetric image (right)

Single-patient dosimetry following 5.4 GBq prescription, absorbed doses to normal organs were calculated to parotid glands (0.9 Gy), submandibular glands (0.8 Gy), right kidney [single functional] (2.6 Gy), liver (0.5 Gy), and spleen (0.5 Gy) (Fig. 6). Total photopeak counts acquired per bed position, (skull, thorax, and pelvis) was: (2.9E6, 8.0E6, 8.0E6) at 4 h, (1.22E6, 3.9E6, 4.7E6) at 24 h, and (0.3E6, 1.0E6, 1.7E6) at 120 h post-administration.

## Discussion

The observed sensitivity appears to be moderated by a mixture of factors relating to the in-field activity which imparts nonlinear effects due to uncorrected dead-time at very high activity levels as well as the local count density. The latter yields an amplification in sensitivity at low counts which has implications for scoring uptake in low-avidity tissues such as bone marrow and may preclude measurements such as whole-body retention over multiple bed step locations; an effect which is more pronounced at lower activity levels as might be required for dosimetric scans several days after therapeutic administration. Additionally, the presence of ^160^Tb impurity may influence the sensitivity of delayed imaging if the time between end-of-separation in production and SPECT image acquisition are of significant time difference. Given ^160^Tb emits an ~ 86 keV gamma with > 13% probability, there is potential for counts detected in both the photopeak and upper scatter window to increase over time relative to the fraction of impurity. This situation, however, is unlikely to manifest in the clinical environment due to radionuclide impurity release limits and the relatively short typical time delay from radionuclide production to post-therapy patient SPECT imaging.

With these potential shortcomings in mind, the stability of scanner sensitivity and RC for avid subregions suggests that post-therapy imaging would be suitable for measuring pharmacokinetics to high-avidity at-risk organs or tumour regions.

Previous work by Marin et al. showed that employing a lower energy photopeak (48.9 keV) is inferior to the 75.4 keV photopeak due to unavoidable artefacts in the reconstruction image. Imaging at this lower photopeak using current gamma camera technology includes several other peaks in the gamma spectrum (Additional file 1: Fig. S4) creating potential sources of quantitative inaccuracy due to attenuation correction from these merged gamma peaks which may further jeopardise the quantitative accuracy of the corrected image. Similar artefacts were not observed during this calibration work. Whilst our work focused on evaluating the linearity of reconstructed counts, future investigation of optimised image reconstruction updates (number of iterations & subsets) and degree of quantitative effect following post-process Gaussian smoothing (see Additional file 1: Fig. S3) are warranted to maximise visual detectability and quantification of small lesions.

Further evaluation of appropriate attenuation correction to account for *k*-edge effects in the energy range below 100 keV may be warranted to achieve optimal image quality and sensitivity in the presence of anatomical material differences or metal implants; however, in this work no apparent uniformity bias was observed with application of Chang’s attenuation correction method [23] in the vendor reconstruction. The energy separation between 75 and 49 keV photopeaks was sufficient to enable both primary photopeak and lower energy scatter windows to be applied without contamination from the lower photon yield. These suggest that existing quantitative SPECT conversion workflows as used with other therapeutic radionuclides will be suitable for use with ^161^Tb after determination of an appropriate, scanner-dependent sensitivity factor.

Contrast recovery and overall image quality was comparable to values obtained when imaging with ^177^Lu [24, 25]. Based on observed RC (Fig. 3) and initial patient dosimetric imaging (Fig. 6), reliable lesion and normal organ dosimetry using ^161^Tb should be feasible for regions larger than 1–2 cm. Again, existing dosimetry workflows—and their potential spatial limitations—should be considered suitable for ^161^Tb. Dosimetric emission properties are similar in terms of path length to ^177^Lu with an amplification of approximately 35% higher absorbed dose per decay at SPECT resolution. It is notable that the biological implications of very short range Auger emission cannot be appreciated in the context of image-based dosimetry as the range of their effects are too short to untangle at SPECT resolution. These nanoscale contributions will need to be inferred based on the known proximity of the targeting molecule to nuclear DNA or based on empirical studies that define a dose–response relationship for a specific ^161^Tb radiopharmaceutical.

## Conclusions

We have shown that quantitative calibration of ^161^Tb is possible with serial SPECT/CT image acquisition over a range of radioactivity levels. Recovery coefficients obtained from ^161^Tb SPECT/CT imaging were comparable to that of ^177^Lu. An amplification in sensitivity at low in-field count rate was observed and may be attributable to reconstruction technique. Quantitative SPECT/CT calibration of ^161^Tb will enable future clinical dosimetric studies.

### Supplementary Information

**Additional file 1**. **Figure S1**: VOI placement of NEMA spheres and two cylindrical regions (turquoise) used as background measurement. **Figure S2**: Example distribution of background VOI counts over a range of activity concentration levels (0.011, 0.088, and 0.618 MBq/mL) and scan durations (2, 8, and 16 seconds). **Figure S3**: NEMA sphere RC and CNR as a function of post-processing Gaussian smoothing from single acquisition with 1.2 GBq in FOV with 8 second frame duration. **Figure S4**: Gamma spectrum obtained from Siemens Intevo-BOLD gamma camera with 990 MBq in NEMA IEC NU2 phantom over 2-minute analyser acquisition. Vertical lines indicate peaked energy windows (red = photopeak [64-79 keV], yellow = lower scatter [60-64 keV], blue = upper scatter [79-83 keV]).

## Data Availability

Not applicable.

## Abbreviations

QSPECT: Quantitative single-photon emission computed tomography
RNT: Radionuclide therapy
DEW: Dual-energy window
TEW: Triple-energy window

## References

[1] Jackson PA, Beauregard J-M, Hofman MS, Kron T, Hogg A, Hicks RJ (2013). An automated voxelized dosimetry tool for radionuclide therapy based on serial quantitative SPECT/CT imaging. Med Phys.

[2] Violet J, Jackson P, Ferdinandus J, Sandhu S, Akhurst T, Iravani A (2019). Dosimetry of 177Lu-PSMA-617 in metastatic castration-resistant prostate cancer: correlations between pretherapeutic imaging and whole-body tumor dosimetry with treatment outcomes. J Nucl Med.

[3] Kennedy J, Chicheportiche A, Keidar Z (2022). Quantitative SPECT/CT for dosimetry of peptide receptor radionuclide therapy. Semin Nucl Med.

[4] Ljungberg M, Celler A, Konijnenberg MW, Eckerman KF, Dewaraja YK, Sjögreen-Gleisner K (2016). MIRD pamphlet No. 26: joint EANM/MIRD guidelines for quantitative 177Lu SPECT applied for dosimetry of radiopharmaceutical therapy. J Nuclear Med.

[5] Jackson PA, Hofman MS, Hicks RJ, Scalzo M, Violet J (2020). Radiation dosimetry in 177Lu-PSMA-617 therapy using a single posttreatment SPECT/CT scan: a novel methodology to generate time- and tissue-specific dose factors. J Nucl Med.

[6] Delker A, Schleske M, Liubchenko G, Berg I, Zacherl MJ, Brendel M (2023). Biodistribution and dosimetry for combined 177Lu-PSMA-I&T/225Ac-PSMA-I&T therapy using multi-isotope quantitative SPECT imaging. Eur J Nucl Med Mol Imaging.

[7] Durán MT, Juget F, Nedjadi Y, Bochud F, Grundler PV, Gracheva N (2020). Determination of 161Tb half-life by three measurement methods. Appl Radiat Isot.

[8] Juget F, Talip Z, Buchillier T, Durán MT, Nedjadi Y, Desorgher L (2021). Determination of the gamma and X-ray emission intensities of terbium-161. Appl Radiat Isot.

[9] Dong J, Bai T, Hu Y, Zhang X, Fan J, Dai Y (2023). Determination of the half-life of 161Tb. Appl Radiat Isot.

[10] Marin I, Rydèn T, Van Essen M, Svensson J, Gracheva N, Köster U (2020). Establishment of a clinical SPECT/CT protocol for imaging of 161Tb. EJNMMI Phys.

[11] Hindié E, Zanotti-Fregonara P, Quinto MA, Morgat C, Champion C (2016). Dose deposits from 90Y, 177Lu, 111In, and 161Tb in micrometastases of various sizes: implications for radiopharmaceutical therapy. J Nucl Med.

[12] Alcocer-Ávila ME, Ferreira A, Quinto MA, Morgat C, Hindié E, Champion C (2020). Radiation doses from 161Tb and 177Lu in single tumour cells and micrometastases. EJNMMI Phys.

[13] Champion C, Quinto MA, Morgat C, Zanotti-Fregonara P, Hindié E (2016). Comparison between three promising ß-emitting radionuclides, (67)Cu, (47)Sc and (161)Tb, with emphasis on doses delivered to minimal residual disease. Theranostics.

[14] Bernhardt P, Svensson J, Hemmingsson J, van der Meulen NP, Zeevaart JR, Konijnenberg MW (2021). Dosimetric analysis of the short-ranged particle emitter 161Tb for radionuclide therapy of metastatic prostate cancer. Cancers.

[15] Müller C, Reber J, Haller S, Dorrer H, Bernhardt P, Zhernosekov K (2014). Direct in vitro and in vivo comparison of 161Tb and 177Lu using a tumour-targeting folate conjugate. Eur J Nucl Med Mol Imaging.

[16] Müller C, Umbricht CA, Gracheva N, Tschan VJ, Pellegrini G, Bernhardt P (2019). Terbium-161 for PSMA-targeted radionuclide therapy of prostate cancer. Eur J Nucl Med Mol Imaging.

[17] Gracheva N, Müller C, Talip Z, Heinitz S, Köster U, Zeevaart JR (2019). Production and characterization of no-carrier-added 161Tb as an alternative to the clinically-applied 177Lu for radionuclide therapy. EJNMMI Radiopharm Chem.

[18] Talip Z, Favaretto C, Geistlich S, van der Meulen NP (2020). A step-by-step guide for the novel radiometal production for medical applications: case studies with 68Ga, 44Sc, 177Lu and 161Tb. Molecules.

[19] Baum RP, Singh A, Kulkarni HR, Bernhardt P, Rydén T, Schuchardt C (2021). First-in-humans application of 161Tb: a feasibility study using 161Tb-DOTATOC. J Nucl Med.

[20] Buteau JP, Kostos LK, Alipour R, Jackson P, McIntosh L, Emmerson B (2023). VIOLET: a phase I/II trial evaluation of radioligand treatment in men with metastatic castration-resistant prostate cancer with 161Tb-PSMA-I&T. J Clin Oncol.

[21] Lowekamp BC, Chen DT, Ibáñez L, Blezek D (2013). The Design of SimpleITK. Front Neuroinform.

[22] Jan S, Santin G, Strul D, Staelens S, Assié K, Autret D (2004). GATE: a simulation toolkit for PET and SPECT. Phys Med Biol.

[23] Chang L-T (1978). A method for attenuation correction in radionuclide computed tomography. IEEE Trans Nucl Sci.

[24] Tran-Gia J, Denis-Bacelar AM, Ferreira KM, Robinson AP, Calvert N, Fenwick AJ (2021). A multicentre and multi-national evaluation of the accuracy of quantitative Lu-177 SPECT/CT imaging performed within the MRTDosimetry project. EJNMMI Phys.

[25] Beauregard J-M, Hofman MS, Pereira JM, Eu P, Hicks RJ (2011). Quantitative 177 Lu SPECT (QSPECT) imaging using a commercially available SPECT/CT system. Cancer Imaging.

