# Supplementary material for: Quantitative calibration of Tb-161 SPECT/CT in view of personalised dosimetry assessment studies
Source: EJNMMI Phys. 2024 Feb 19;11:18. doi: 10.1186/s40658-024-00611-9 (PMC10876500; doi:10.1186/s40658-024-00611-9)
Supplement: Supplementary file 1 — Additional file 1. Figure S1: VOI placement of NEMA spheres and two cylindrical regions (turquoise) used as background measurement. Figure S2: Example distribution of background VOI counts over a range of activity concentration levels (0.011, 0.088, and 0.618 MBq/mL) and scan durations (2, 8, and 16 seconds). Figure S3: NEMA sphere RC and CNR as a function of post-processing Gaussian smoothing from single acquisition with 1.2 GBq in FOV with 8 second frame duration. Figure S4: Gamma spectrum obtained from Siemens Intevo-BOLD gamma camera with 990 MBq in NEMA IEC NU2 phantom over 2-minute analyser acquisition. Vertical lines indicate peaked energy windows (red = photopeak [64-79 keV], yellow = lower scatter [60-64 keV], blue = upper scatter [79-83 keV]). [file 40658_2024_611_MOESM1_ESM.docx]

**SUPPLEMENTARY FIGURES:**


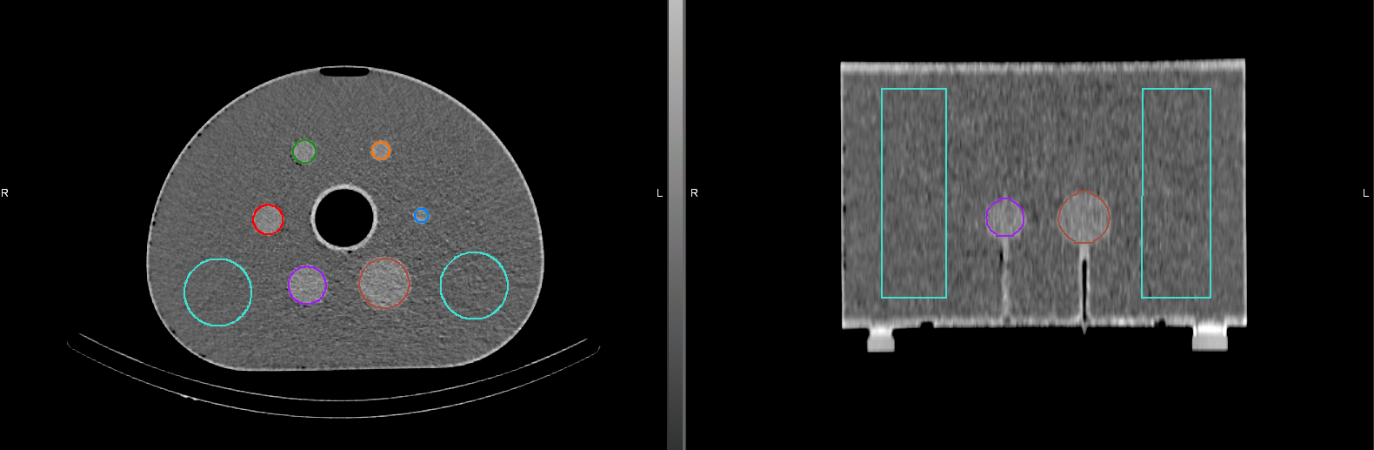


Supplementary Figure 1 — VOI placement of NEMA spheres and two cylindrical regions (turquoise) used as background measurement


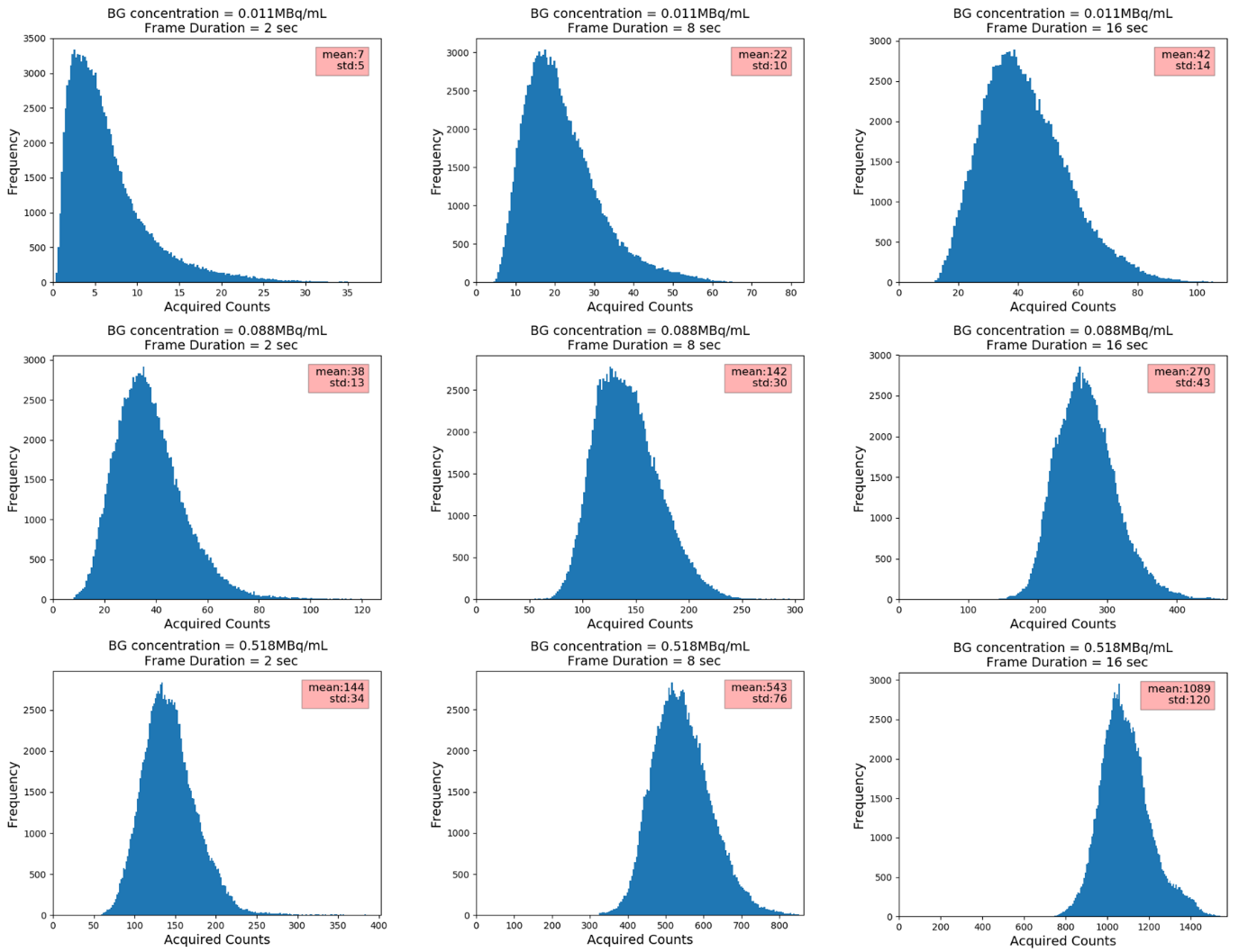


Supplementary Figure 2 — Example distribution of background VOI counts over a range of activity concentration levels (0.011, 0.088, and 0.618 MBq/mL) and scan durations (2, 8, and 16 seconds).


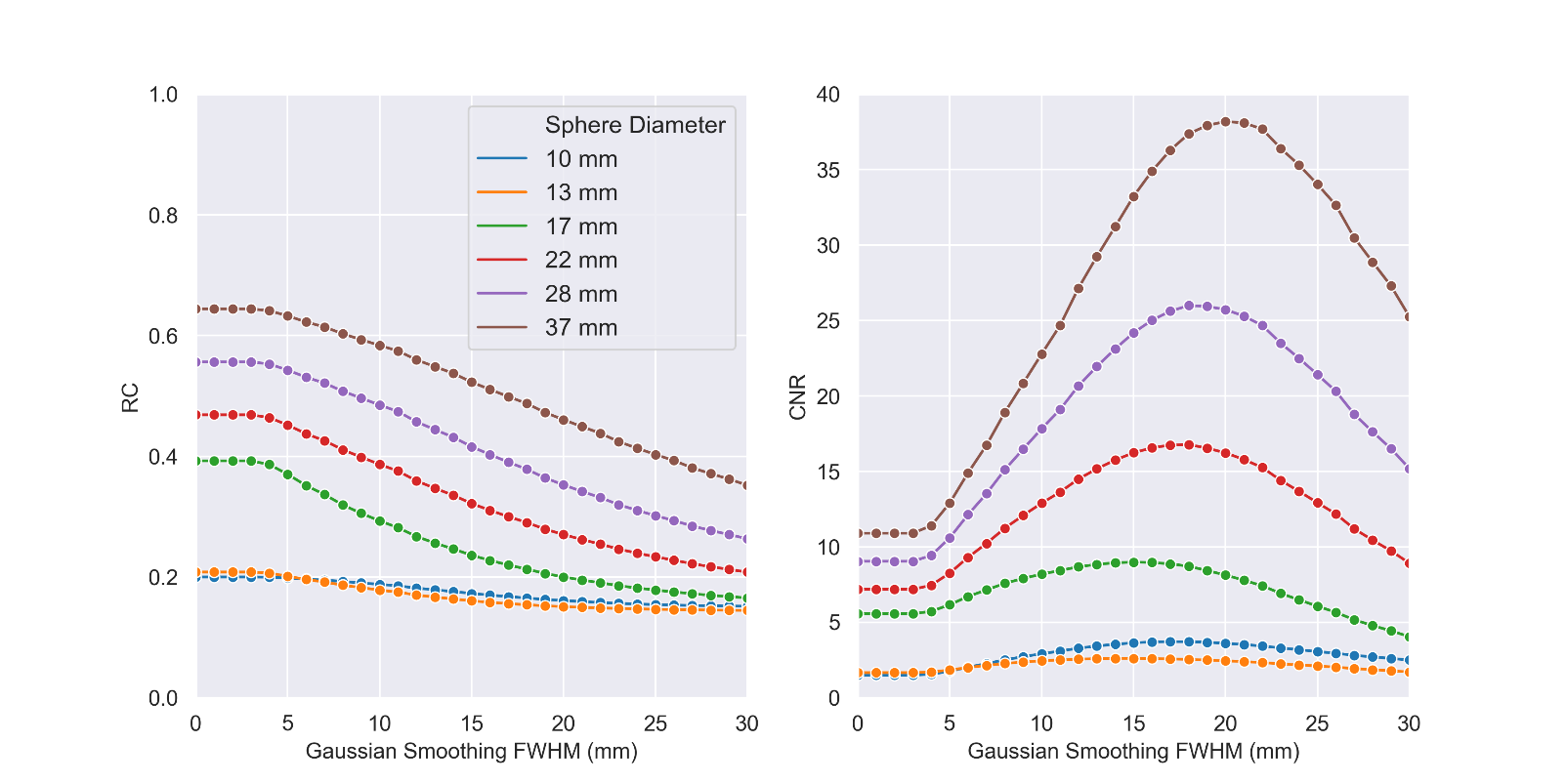


Supplementary Figure 3 — NEMA Sphere RC and CNR as a function of post-processing gaussian smoothing from single acquisition with 1.2 GBq in FOV with 8 second frame duration.


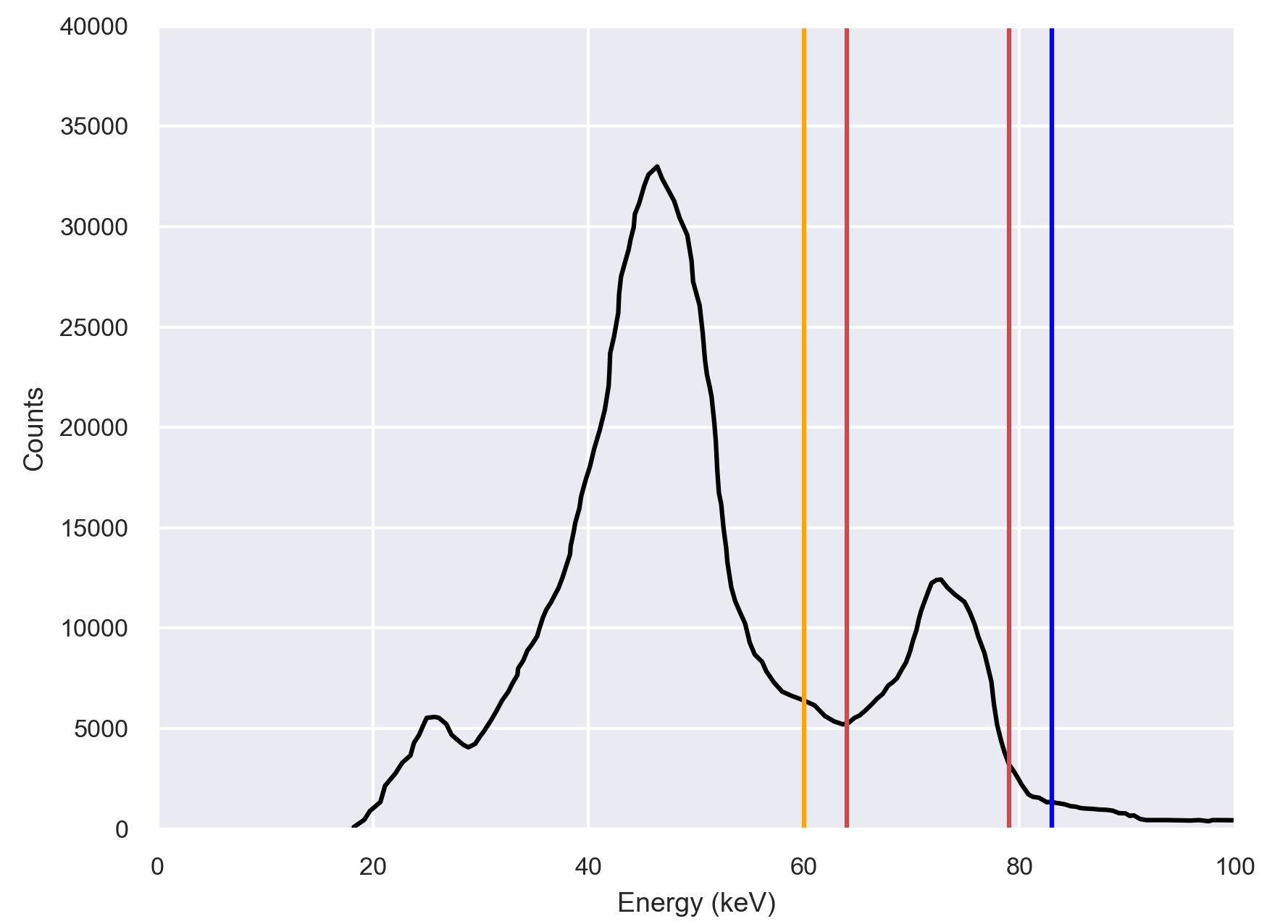


Supplementary Figure 4 — Gamma spectrum obtained from Siemens Intevo-BOLD gamma camera with 990 MBq in NEMA IEC NU2 phantom over 2-minute analyser acquisition. Vertical lines indicate peaked energy windows (red = photopeak [64-79 keV], yellow = lower scatter [60-64 keV], blue = upper scatter [79-83 keV])
